# Canine Transmissible Venereal Tumor: Anatomical Locations, Chemotherapy Response, and Epidemiological Aspects at a Veterinary Teaching Hospital in Brazil (2012–2022)

**DOI:** 10.3390/ani15121675

**Published:** 2025-06-06

**Authors:** Pedro Antônio Bronhara Pimentel, Lorena Diniz Macedo Silva Maia, Isadora Maria Sátiro de Oliveira, Camila Stefanie Fonseca de Oliveira, Antonio Giuliano, Angel Almendros, Rodrigo dos Santos Horta

**Affiliations:** 1 Department of Veterinary Medicine and Surgery, Veterinary School, Universidade Federal de Minas Gerais (UFMG), Belo Horizonte 31270-901, MG, Brazil; pedrobpimentel@gmail.com; 2 Department of Preventive Veterinary Medicine, Veterinary School, Universidade Federal de Minas Gerais (UFMG), Belo Horizonte 31270-901, MG, Brazil; lorena.macedo@yahoo.com.br (L.D.M.S.M.); sfo.camila@gmail.com (C.S.F.d.O.); 3 Department of Morphology, Institute of Biological Sciences, Universidade Federal de Minas Gerais (UFMG), Belo Horizonte 31270-901, MG, Brazil; isadorasatiro@gmail.com; 4 Harvest Veterinary Oncology Center (HVOC), Kwai Fong, Kowloon, Hong Kong; agiulian@cityu.edu.hk; 5 Department of Veterinary Clinical Sciences, The Jockey Club College of Veterinary Medicine and Life Sciences, City University of Hong Kong, Kowloon, Hong Kong

**Keywords:** sticker tumor, contagious, neoplasm, vincristine, round cell tumor

## Abstract

Canine transmissible venereal tumor (CTVT) is a contagious neoplasm with low metastatic potential, primarily affecting free-roaming and stray dogs. Despite its global presence, epidemiological data from some regions remain limited. This study employed a retrospective observational design and analyzed 131 CTVT cases diagnosed via cytology or histopathology at a veterinary teaching hospital in Belo Horizonte, Brazil, aiming to describe the anatomical distribution, treatment outcomes, and epidemiological patterns. Most affected dogs were mixed-breed (70.2%) and female (61.1%), with a median age of 4.5 years. Genital involvement was most common (87.0%), followed by cutaneous (10.7%), nasal (6.1%), and oral (4.6%) tumors. Concurrent tumor locations included genital-cutaneous (5.3%) and oronasal (3.1%). Females had more genital cases, while males were more likely to present cutaneous and nasal CTVT, with 5.2 times greater odds for nasal tumors (OR = 5.2; 95% CI = 1.2–25.9). Purebred dogs also had increased odds of nasal involvement (OR = 8.2; 95% CI = 1.9–40.7). Vincristine chemotherapy was effective, and the number of sessions required for a complete response was not associated with clinical presentation, breed or size. These findings highlight the varied presentations of CTVT and reinforce the need for clinical awareness of non-genital forms.

## Simple Summary

Canine transmissible venereal tumor (CTVT) is a contagious cancer that spreads through direct contact between dogs (mainly during mating). It is more common in countries with many stray or free-roaming dogs, but there is still limited information about how it behaves in different regions. This study aims to describe how this tumor appears in dogs treated at a veterinary teaching hospital in Brazil, identify risk factors such as sex and breed, examine where in the body the tumor occurs, and evaluate how well the standard treatment works. It also explored whether the location of the tumor was related to these risk factors. Records from 131 dogs were reviewed, most of them being mixed-breed and female. While the tumor was most often found in the genital area, it also appeared on the skin, in the nose, and in the mouth. Male and purebred dogs were more likely to have tumors in the nose. All dogs received a common chemotherapy drug, which worked well in most cases, regardless of the dog’s size, breed, or tumor location. These findings contribute to the epidemiological understanding of CTVT in Brazil, which may support future efforts to improve disease monitoring and control, especially in areas with limited resources and large populations of free-roaming dogs.

## 1. Introduction

Canine transmissible venereal tumor (CTVT) is a clonally transmissible malignant neoplasm that originated approximately 6000 years ago and is maintained through the direct transmission of somatic cells between dogs [1]. In recent decades, it has been studied as a cancer research model to understand neoplastic progression and survival mechanisms [2]. Its contagious biological behavior allows for distinct prevention strategies compared to most cancers in domestic animals. In regions with strict population control campaigns, such as Western European countries, autochthonous cases have been nearly eradicated [3,4,5]. In contrast, CTVT is endemic in most low-income countries, particularly Latin America, Africa, Eastern Europe, and Asia [6,7,8,9,10,11].

Treatments for CTVT are standardized and primarily consist of vincristine monotherapy, which has a high response rate, while surgery should be considered only in cases of chemoresistance [12,13]. Risk factors include free-roaming status and low neutering rate, as the primary transmission is sexual [1,4]. In Brazil, the tumor remains widely disseminated due to the persistence of these risk factors within canine populations [14,15,16].

The most common anatomical location of CTVT is the genitalia, involving the vulva and vagina in females and the penis and prepuce in males. Nevertheless, cutaneous, nasal, and oral forms also occur through implantation [13,14]. While genital manifestations have been well-documented in the literature for over a century [17,18,19], atypical non-genital forms have historically been considered rare and only described in greater detail in recent decades [9,15,20]. Despite its low metastatic potential, CTVT can spread to lymph nodes, though this occurs in fewer than 5% of cases [13]. In Minas Gerais, Brazil, most previously reported CTVT cases involved the genital form, which may not fully represent the broader clinical presentation of the disease.

This study aims to clinically characterize dogs with CTVT treated at a veterinary teaching hospital in Brazil, identifying epidemiological characteristics, anatomical locations of the tumor, and treatment efficacy. Additionally, the study investigated the potential correlation of CTVT anatomical location with epidemiological aspects.

## 2. Materials and Methods

### 2.1. Data Collection and Preprocessing

This study employed a retrospective observational design, analyzing medical records of dogs diagnosed with CTVT at the veterinary teaching hospital (VH) at Universidade Federal de Minas Gerais, in Belo Horizonte, Brazil, between 2012 and 2022. Clinical, demographic, and treatment-related data were collected and statistically analyzed to identify epidemiological trends and anatomical tumor locations. Patient information included age at diagnosis, year of diagnosis, sex, breed, clinical manifestations of the disease (genital, ocular, nasal, oral, cutaneous, perianal, in lymph nodes, and other less frequent sites), and treatment protocol, including chemotherapy and the number of sessions. CTVT diagnosis was confirmed by morphological examination, cytology, or histopathology. Age was recorded at the time of diagnosis. Since many CTVT-affected dogs were rescued, determining their exact age was challenging. These patients were excluded from the age analysis and reported in Supplementary Materials S1 as “undetermined”. Age and body size were classified into distinct categories based on established criteria [21]. Dogs were categorized as either young (≤6 years) or old (>6 years). Additionally, they were classified by size as small breed (<15 kg), medium breed (15–25 kg), or large breed (>25 kg).

Regarding the treatment, vincristine sulfate was administered intravenously at 0.75 mg/m^2^ weekly for 3–7 sessions, with a dosage reduction to 0.5 mg/m^2^ in cases of grade II–IV neutropenia [12,22]. A minimum of three weekly doses was given to achieve complete remission. In cases of chemotherapy resistance, treatment was adjusted by either transitioning to surgical excision or substituting vincristine with doxorubicin or lomustine. Therapeutic interventions were initiated only after definitive diagnostic confirmation. Chemoresistant cases were defined as those that failed to achieve a complete response after six sessions of the conventional vincristine protocol or showed stable disease after four sessions.

Complete remission (CR) was defined as the absence of macroscopic identification of the neoplasm during follow-up appointments after the end of the chemotherapy protocol. Partial remission (PR) was considered a reduction of more than 30% in tumor volume after the chemotherapy protocol, but some of the tumors remained. Stable disease (SD) was defined as a decrease of less than 30% in the tumor volume or an increase of less than 20%. Progressive disease (PD) indicated a greater than 20% increase in tumor volume. An objective response (OR) was defined as the combination of complete response and partial remission. If there was any uncertainty, a cytological examination of the suspected tissue was performed. The criteria are based on the Veterinary Comparative Oncology Group consensus for clinical response criteria for canine solid tumors [22].

The data were extracted from the VH clinical data storage software (SGV) (desktop version) and compiled in Microsoft Excel^®^. The complete dataset is available in the Supplementary Materials S1.

### 2.2. Statistical Analysis

The crude incidence of CTVT was calculated by dividing the number of dogs diagnosed with CTVT by the total number of canine patients seen at the veterinary teaching hospital during the study period, and the result was expressed per 1000 dogs. A chi-squared test was used to assess differences in case frequency between two time periods (2012–2017 and 2018–2022).

The study analyzed demographic and clinical variables, including sex, body weight (categorized by breed size), age, breed, CTVT anatomical locations (genital, cutaneous, nasal, and oral), treatment protocols, and number of chemotherapy sessions. Data were organized in Microsoft Excel^®^ (version 2013), with descriptive statistics summarized in tables and figures. Associations between categorical variables were evaluated using Fisher’s exact test, while differences in proportional distributions were assessed via chi-square, as appropriate. Odds ratios with 95% confidence intervals (CI) were calculated using univariate analyses to quantify association strengths. All analyses were conducted in Stata (version 17.0), with statistical significance set at α = 0.05 [23].

## 3. Results

### 3.1. Epidemiology

**Figure 1:**
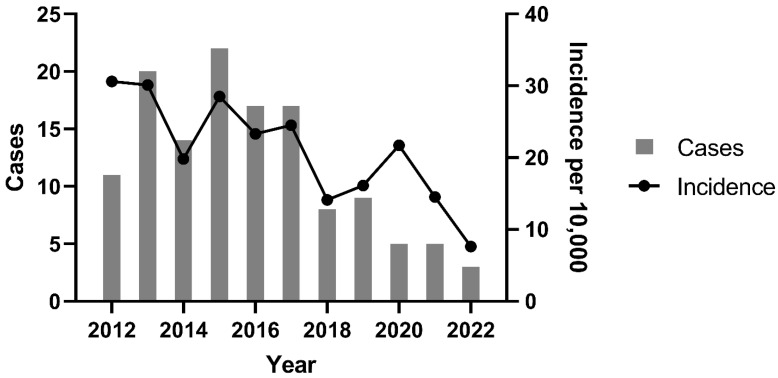
CTVT cases and incidence of CTVT per year between 2012 and 2022 at the Veterinary Hospital of the Universidade Federal de Minas Gerais, Belo Horizonte, Brazil (2021–2022).

Between 2012 and 2022, the VH recorded 60,256 canine patients, with 3020 (5.0%) classified as oncology cases. At the Oncology Service, 131 dogs (4.3% of oncology cases) were diagnosed with CTVT. The crude incidence rate of CTVT within the total clinical population was 2.17 cases per 1000 dogs (0.2%; 131/60,256), equating to 1 case per 459.9 dogs (Figure 1).

**Figure 2:**
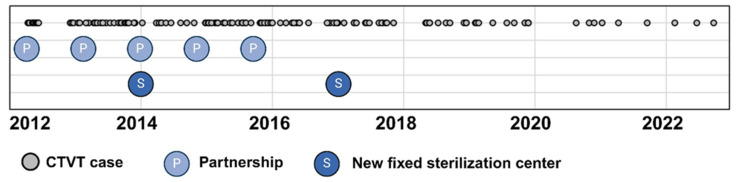
Temporal distribution of CTVT cases at the Veterinary Hospital of the Universidade Federal de Minas Gerais, Belo Horizonte, Brazil (2021–2022). Grey circles: individual CTVT cases; S: newly established fixed canine sterilization centers; P: partnerships between the Zoonosis Surveillance Unit (ZSU) and civil society/nongovernmental organizations (NGOs). Created using BioRender.com.

A significant decline in CTVT incidence occurred between 2012–2017 and 2018–2022 (*p* = 0.004). The peak incidence was observed in 2012 (30.6 cases per 10,000 dogs; 1:327 cases), while the nadir occurred in 2022 (7.6 cases per 10,000 dogs; 1:1317 cases). Temporal analysis revealed a progressive reduction in both annual CTVT diagnoses and incidence rates over the study period. This trend coincided with intensified public canine neutering initiatives. Figure 2 clearly demonstrates the timeline of intervention efforts in relation to the distribution of CTVT cases, including the establishment of fixed sterilization centers (S) and the initiation of partnerships (P) between the Zoonosis Surveillance Unit (ZSU) and nongovernmental organizations (NGOs).

Among all dogs treated at the VH, 44,751 (74.7%) were purebred and 15,269 (25.3%) were mixed-breed. Conversely, in the target population of this study, mixed-breed dogs comprised 70.2% (92/131) of CTVT cases, and purebred dogs represented 29.8% (39/131). This corresponds to an incidence of approximately 8.7 cases per 10,000 purebred dogs and 60.3 cases per 10,000 mixed-breed dogs, suggesting a markedly higher incidence of CTVT among mixed-breed individuals. Purebred CTVT cases included Poodle (6.9%, 9/131), Labrador Retriever (3.8%, 5/131), Boxer (3.1%, 4/131), and Golden Retriever, German Shepherd, Shih Tzu, Miniature Pinscher, and Yorkshire Terrier (2.3% each, 3/131). Other breeds (Basset Hound, Beagle, Chow-Chow, Belgian Shepherd, American Pitbull, English Pointer) accounted for 4.6% (6/131). Mixed-breed dogs exhibited 6.95-fold higher odds of developing CTVT compared to purebred dogs (95% CI = 4.8–10.1; *p* < 0.0001). CTVT cases comprised 80 females (61.1%) and 51 males (38.9%), with no association between sex and the number of chemotherapy sessions required for remission (*p* = 0.743).

A significant association was observed between nasal CTVT location and purebred dogs (*p* = 0.009), with purebreds exhibiting 8.2-fold higher odds compared to mixed-breed dogs (95% CI = 1.9–40.7). Conversely, genital locations were more prevalent in mixed-breed dogs (*p* = 0.04), with mixed-breeds demonstrating 3.2-fold higher odds relative to purebreds (95% CI = 1.1–9.2). No significant associations were identified for lymph node, ocular, perianal, cutaneous, or oral locations (*p* > 0.05 via Fisher’s exact test), as shown in Table 1.

The mean age of dogs diagnosed with CTVT was 4.5 ± 3.1 years. The age distribution was broad, ranging from 6 months to 14 years. Younger animals (≤6 years) accounted for 77.2% (76/98) of cases, and the peak incidence occurred in the 1.1–3-year-old cohort (38.6%, 38/98). Only 7.1% (7/98) of cases were observed in dogs older than 10 years.

### 3.2. Anatomical Locations

**Figure 3:**
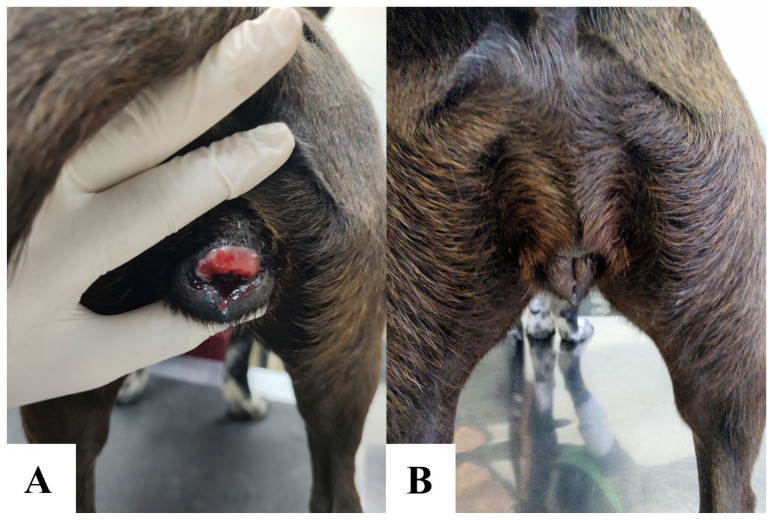
Vulvar canine transmissible venereal tumor in a 1-year-old dog. Pre-treatment vulvar CTVT (**A**). Post-chemotherapy resolution following 4 weekly intravenous vincristine sulfate doses (0.7 mg/m^2^). Complete remission was achieved with no residual macroscopic disease (**B**).

Genital involvement was the most common anatomical location of CTVT, occurring in 87% (114/131) of cases. Exclusive genital lesions—affecting the vulva/vagina in females and penis/prepuce in males—were observed in 77.9% (102/131) of dogs. Concurrent genital and extragenital tumors accounted for 9.2% (12/131) of cases. Figure 3 illustrates a dog with CTVT treated with vincristine sulfate.

Extragenital CTVT occurred in 22.1% (29/131) of cases, with 13% (17/131) presenting exclusively in non-genital sites (cutaneous, nasal, oral, or other anatomical locations). Cutaneous involvement was the most frequent extragenital CTVT (11.5%, 15/131), followed by nasal (6.1%, 8/131) and oral (4.6%, 6/131) locations. Perianal location and lymph node metastasis each accounted for 3.1% (4/131) of cases, both assessed by cytology. Rare locations included ocular (2.3%, 3/131), and single instances of mammary gland, urethral, and vesical involvement.

A unique case exhibited central nervous system (CNS) involvement, with a prosencephalic lesion. Following combined lomustine and vincristine sulfate chemotherapy, computed tomography (CT) confirmed the resolution of the right cerebral lobe nodule, accompanied by a marked improvement in neurological signs. Cytological diagnosis of CTVT had been previously established in the same patient via ocular lesion assessment.

The majority of CTVT cases (84%, 110/131) presented with a single location, while 16% (21/131) exhibited co-occurring sites. Among these, 12.9% (17/131) had two concurrent locations, 2.3% (3/131) had three, and 0.8% (1/131) presented five. The most common co-occurrences were genital-cutaneous (5.3%) and oronasal (3.1%) presentations. No association was observed between canine body size and CTVT locations.

Table 2 summarizes sex-based differences in the anatomical location of CTVT cases. Oronasal location demonstrated the only statistically significant association with sex (*p* = 0.021). Non-significant trends included a female predominance of genital location and a male predominance of exclusive cutaneous location; however, neither reached significance in odds ratio analyses.

Table 3 presents odds ratios for individual CTVT manifestations, with male dogs as the reference category. Males were overrepresented in nasal (75%, 6/8) and oral (66.6%, 4/6) locations. Similarly, cutaneous CTVT occurred more frequently in males (60%, 9/15; *p* = 0.094). While nasal CTVT showed an odds ratio of 5.2 (95% CI = 1.2–25.9), occurring in 6/51 males vs. 2/80 females, the association approached but did not reach statistical significance (*p* = 0.055), suggesting a non-significant trend. No other manifestations demonstrated significant sex-based associations.

### 3.3. Diagnosis

Diagnostic confirmation of CTVT was achieved for all cases. Cytology—the institutional gold standard for CTVT diagnosis—was used in 97.7% (128/131) of cases, while histopathology was reserved for 2.3% (3/131) of patients. All histopathology-confirmed cases presented with extragenital manifestations (two nasal, one ocular), where CTVT was not the initial clinical suspicion.

**Figure 4:**
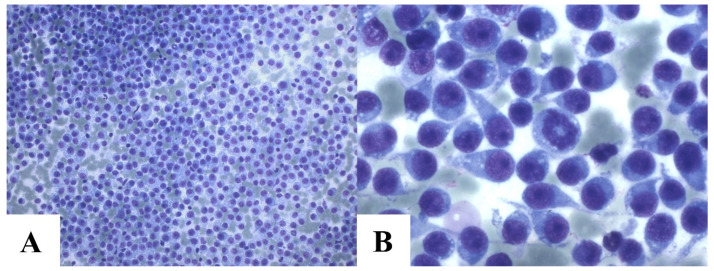
Cytology of canine transmissible venereal tumor. Intense cellularity of CTVT cells. Romanowsky, 20 × 2 magnification (**A**). Intense cellularity of CTVT cells with mitosis and various tadpole cells. Cells present mostly plasmacytic cytomorphology. Romanowsky, 100 × 2 magnification (**B**).

The cytological evaluation revealed characteristic CTVT features, including round to ovoid neoplastic cells with well-defined nuclei, coarse chromatin, a single prominent nucleolus, and cytoplasmic vacuoles. Nuclear-to-cytoplasmic ratios varied, with higher ratios in lymphocytic variants and moderate-to-low ratios in plasmacytic variants (Figure 4). Additional findings included frequent mitotic figures (typical and atypical), “tadpole cells” (tumor cells with cytoplasmic extensions and mesenchymal-like morphology), intralesional bacteria (noted mainly in imprint samples), and mixed inflammatory infiltrates (neutrophils, eosinophils, macrophages, and lymphocytes).

### 3.4. Treatment

The primary treatment modality was vincristine sulfate chemotherapy, administered to 97.7% (85/87) of cases. An objective response (OR) was achieved in 97.6% (83/85) of treated dogs, including 91.8% (78/85) with complete remission (CR) and 5.8% (5/85) with partial remission (PR). Stable disease (SD) occurred in 2.4% (2/85) of cases. In nasal cases, OR was assessed based on the remission of clinical signs and cytological findings, as advanced imaging techniques were not feasible due to economic constraints. The mean number of sessions was 4.5 (median: 4; SD: ±0.97). No association was observed between tumor location and the number of chemotherapy sessions.

For cases with partial remission following vincristine sulfate therapy, secondary interventions included the following: surgical excision (*n* = 3), lomustine monotherapy (50 mg/m^2^ orally, 3 sessions; *n* = 1), and doxorubicin monotherapy (30 mg/m^2^ intravenously, 1 session; *n* = 1). All patients attained complete remission post-intervention. One dog treated with vincristine + lomustine experienced recurrence at 8 months but achieved CR after retreatment with the same protocol.

Two patients with stable disease following vincristine sulfate therapy underwent surgical excision, achieving sustained remission without recurrence. Separately, two additional patients underwent primary surgical excision prior to CTVT diagnosis (due to initial diagnostic uncertainty), with no recurrence observed post-intervention. Additional case-specific details are provided in the Supplementary Materials S1.

## 4. Discussion

Among the most prevalent canine neoplasms in Latin America, CTVT has maintained endemic status across diverse regions for decades due to persistent risk factors such as free-roaming and entire dog populations [8,24,25]. This study provides critical epidemiological insights into the disease, offering the first longitudinal analysis of its incidence over a 10-year period (2012–2022) in Belo Horizonte, Brazil. This retrospective study identified a CTVT incidence rate of 217.4 cases per 100,000 dogs (2012–2022), a metric underrepresented in Brazilian literature. Reported frequencies of CTVT as a proportion of all canine tumors vary widely (0.9–17.1%) across prior studies, with diagnostic methodology critically influencing these estimates [26,27,28,29,30]. For example, histopathology-based surveys (e.g., São Paulo: 2% frequency, HDI 0.805) yield lower rates than cytology-driven analyses (e.g., Londrina: 11.9% frequency, HDI 0.778), despite comparable regional socioeconomic profiles [31]. These discrepancies underscore the necessity of standardized diagnostic protocols to improve epidemiological accuracy and cross-study comparability.

While CTVT is now most prevalent in low-income regions, historical records highlight its formerly widespread global distribution. In 1968, it was described as the most common canine tumor in Jamaica [32], and Murray [33] reported a CTVT frequency of 12.4% (20/161) in Kenya. Nowadays, CTVT remains highly prevalent in stray dog populations, particularly in countries like Mexico, where it persists as the most frequent canine neoplasm [34]. Population bias significantly impacts reported incidence rates: studies from veterinary hospitals specializing in referral cases or high-cost treatments likely underrepresent CTVT prevalence due to socioeconomic barriers limiting access for stray or low-income-owned dogs. Such settings may inadvertently exclude the populations most vulnerable to CTVT, skewing data toward owned, insured, or urban canine cohorts.

A marked decline in CTVT incidence has been well-documented in high-income regions such as the United Kingdom, where comprehensive dog population control policies have rendered the disease rare for decades [4,5]. Similarly, our study observed a decreasing trend at this particular veterinary teaching hospital in Belo Horizonte, Brazil, though cautious interpretation is warranted given the hospital-based cohort’s inherent limitations. To robustly assess CTVT epidemiology in this region, future investigations should prioritize population-wide sampling, encompassing both owned and free-roaming dogs, including all hospitals, to mitigate referral bias and socioeconomic confounding factors.

The observed decline in CTVT incidence may reflect the successful disruption of its transmission dynamics through targeted interventions, as evidenced by global precedents [5]. In Belo Horizonte, key initiatives include expanded neutering programs since 2006, an increase in the number of fixed centers for canine sterilization from one in 2007 to five in 2019, the implementation of mobile sterilization units in 2008, and the establishment of public–NGO partnerships between 2011 and 2015 [35,36]. While direct data on stray dog populations remain limited, these measures likely reduced high-risk, free-roaming cohorts—a critical reservoir for CTVT transmission.

Genital location predominated in this cohort, consistent with prior studies reporting CTVT as a sexually transmitted neoplasm primarily localized to genital organs [13,37]. Frequencies in the literature align closely with our findings, ranging from 85.7% [13] to 87% [13]. However, some reports describe even higher genital presentation rates (>95%), a discrepancy potentially attributable to the underdiagnosis of extragenital cases [15]. The disease’s nomenclature—emphasizing its venereal transmission—may inadvertently bias clinical suspicion toward genital presentations, leading to delayed or missed diagnoses of atypical forms [38,39].

The frequency of extragenital locations varies widely among studies, and some, such as cutaneous and lymph node involvement, are not mentioned in some surveys [16,38]. Cutaneous presentation was the most frequent extragenital site reported in this study, as previously demonstrated by Peixoto et al. in 2016 [15], with 21.7% of cases affecting the skin. Extragenital locations, including nasal and oral manifestations, tend to be more frequent after cutaneous involvement, as suggested by the literature, and they are more common than other sites and lymph node metastases [13,15,16].

Although the majority of dogs diagnosed with CTVT are females (61.1%), there is an inversion of this proportion when analyzing the oral and nasal locations of the disease. In this context, males accounted for 75% (6/8) and 66.6% (4/6) of dogs affected by nasal and oral sites, respectively. A similar pattern was observed for cutaneous manifestations, in which males presented a slightly higher frequency than females, representing 60% (9/15) of the cases. While these findings may suggest a potential behavioral predisposition—such as sniffing and licking habits—among males [40,41], the number of cases in these subgroups was small, and the differences were not statistically significant. Further studies with larger sample sizes and behavioral assessments are needed to explore possible sex-based predispositions in extragenital and concurrent CTVT presentations.

A sexual predisposition for CTVT was not indicated, but there were more females affected than males in this survey, a trend also reported in most studies evaluating this sex ratio [39] and very close to what was identified by Araújo et al., 2016 [13]. If age, sex, and other risk factors were separately analyzed in order to see clinical patterns of the disease, this could be better understood.

The average age of dogs with this neoplasm appeared to be older than what was reported in the previous studies [26,42], but aligns with recent literature [37,43], possibly due to the increased life expectancy of companion animals. Recent studies have shown that mixed-breed dogs are the most commonly affected by the neoplasm [37,44], which can be explained by their contact with the outdoor environment, either before adoption or even while under the care of owners.

The dogs’ breed was analyzed as a risk factor related to clinical manifestations in this and previous studies [11,15]. Lifestyle factors, such as the likelihood of mixed-breed dogs roaming more freely than purebred dogs, could significantly influence the risk of contracting CTVT. While lifestyle categorization was not available in the collected data and cannot be gathered retrospectively, a logical association can be established: owned dogs may have a higher risk of developing oral/nasal/perianal/cutaneous CTVT due to licking behaviors, a non-classical dissemination route, whereas free-roaming dogs may have a higher risk of developing genital CTVT, which aligns with the classic mode of disease dissemination [9,41].

The occurrence of CTVT concurrent locations might complicate the diagnosis and is poorly mentioned in Brazil. Recently, in Rio de Janeiro [45], it was demonstrated the occurrence of associated sites in 7.9% (20/252) of cases, which is much greater than the figure reported by Peixoto et al., 2016 [15] and in Rio de Janeiro, where concurrent genital and extragenital locations occurred in only 1.9% of cases. Conversely, most previous studies did not mention this occurrence of two or more sites [19,44,46]. In Grenada, about 11.5% (9/78) of CTVT cases involved two or more manifestations [47]. A retrospective study involving dogs with an ocular presentation of the tumor in Greece revealed that 12% (3/25) of patients had another concurrent site, with 4% (1/25) being genital and 8% (2/25) oronasal [48]. The most representative study of CTVT in the United States in recent decades indicated the presence of multiple manifestations in 6.9% (2/29) of dogs between 1984 and 1996 [49]. In dogs with canine visceral leishmaniasis in Italy, 21% (4/19) of individuals developed cutaneous tumors concurrently with genital sites, possibly indicating a relationship with protozoan-mediated immunosuppression affecting CTVT, although the sample size was limited [50]. Our study showed a high frequency of concurrent presentations (14.5%), which was greater than the frequencies reported in Brazilian studies and similar to frequencies reported in other geographical locations [44,45,47]. This is possibly due to historical underreporting and limited recognition of extragenital CTVT. In previous reports from the same state, Minas Gerais, the prevalence of extragenital presentations was as low as 4%, with no cases of concurrent anatomical involvement reported among 144 dogs [19].

Cytology was the primary method for diagnosing CTVTs, consistent with previous studies on this neoplasm [29,37]. It is a faster and more cost-effective method than histopathology and is commonly used for diagnosing round-cell tumors [51,52]. In contrast, histopathology is primarily employed for morphological diagnosis in necropsies, especially for tumors with atypical manifestations or for examination following surgical excision [27,53]. This was the case in our study, where CTVT was diagnosed by histopathology following an excisional biopsy when it was not the primary clinical suspicion.

The standard treatment with vincristine sulfate showed an expected high efficacy, previously reported worldwide [12,54,55]. CTVT is a chemosensitive neoplasm, and spontaneous regression, even if partial and not long-lasting, can occur more frequently than in dogs with other neoplasms [45,56,57], primarily due to the potential immune recognition of tumor antigens after therapy, as demonstrated through the use of dendritic cells pulsed with tumor exosomes [58]. The use of other chemotherapeutic agents, such as lomustine, is reserved for resistant cases to vincristine [59]. Surgery as a treatment typically results in high recurrence rates [60,61]. However, in this study, there were no instances of recurrence among the patients who underwent surgery. Therefore, it was only recommended when the tumor was chemoresistant or when there was another surgical indication, such as enucleation or resolution of peripheral neuropathy.

The limitations of this study include the dropout of patients after complete remission of the neoplasm, which hinders medium and long-term oncological follow-up. However, recurrences after conventional vincristine therapy are uncommon. Additionally, this neoplasm more frequently affects stray dogs, which are associated with financial restrictions due to socioeconomic demographics, which can make treatment costs unfeasible, favoring postdiagnosis evasion. A major limitation of this study from an epidemiological standpoint is that the data were derived from a single veterinary hospital. As such, the findings cannot be generalized to represent the true incidence of CTVT in the broader region. Given that there are likely hundreds of veterinary clinics and hospitals in the area—each potentially seeing varying case numbers and patterns—the incidence reported here may not accurately reflect the regional epidemiological profile of the disease. While univariate analyses provided valuable insights, multivariate approaches would better control for confounders and clarify relationships between sex, breeds, and the anatomical location of CTVT.

## 5. Conclusions

This study identifies mixed-breed dogs as the most affected by CTVT, and those were more likely to develop genital involvement. Meanwhile, purebred dogs exhibited an increased risk for nasal presentations. Vincristine sulfate demonstrated a high complete remission rate, reinforcing its role as the gold-standard therapy.

Brazi is confirmed as an endemic country for CTVT. Although the incidence declined over the decade—likely due to neutering campaigns and public health interventions—the disease may persist in free-roaming populations. The anatomical locations of CTVT align with global trends; however, underdiagnosis of atypical cases remains a concern. To address this issue, standardized diagnostic protocols and expanded surveillance in understudied regions are essential for refining control strategies and reducing CTVT’s global burden.

## Figures and Tables

**Table 1:** Associations between dog breed and canine transmissible venereal tumor locations at the Veterinary Hospital of the Universidade Federal de Minas Gerais, Belo Horizonte, Brazil (2012–2022).

CTVT Anatomical Locations	Purebred		Mixed-Breed		Total	*p*-Value	Odds Ratio (95% CI) ^1^
	*n*	Incidence	*n*	Incidence			
Genital	30	1:1525.0	84	1:181.8	114	0.043 *	0.3 (0.1–0.9)
Lymph nodes	0	–	4	1:3817.3	4	0.317	– ^2^
Ocular	0	–	3	1:5089.7	3	0.554	– ^2^
Perianal	2	1:22,875.5	2	1:7634.5	4	0.582	2.4 (0.4–15.9)
Cutaneous	5	1:9150.2	10	1:1526.9	15	0.768	1.2 (0.4–3.7)
Nasal	6	1:7625.2	2	1:7634.5	8	0.009 *	8.2 (1.9–40.7)
Oral	2	1:22,875.5	4	1:3817.3	6	1.0	1.2 (0.2–5.3)
Total	39	1:1147.5	92	1:165.9	131	<0.0001 *	0.1 (0.1–0.2)

* Significative association in Fisher’s exact test (*p* < 0.05). ^1^ Odds ratio considered purebred as referent category (exposed). ^2^ In these binary outcomes in which the purebred group had no occurrences of the outcome, calculating the odds ratio was not appropriate. The sum of cases does not correspond to the sum of cases for each location due to cases with concomitant sites.

**Table 2:** Number and type of anatomical locations of canine transmissible venereal tumor at the Veterinary Hospital of the Universidade Federal de Minas Gerais, Belo Horizonte, Brazil (2012–2022).

Anatomical Locations		Cases in Male Dogs	Cases in Female Dogs	Total of Cases	Frequency (%) in Male Dogs	Frequency (%) in Female Dogs	Total Frequency (%)	*p*-Value	Odds Ratio ^1^
Locations per dog									
	1	40/51	70/80	110/131	78.4%	87.5%	83.9%	0.222	0.5 (0.2–1.3)
	2	8/51	9/80	17/131	15.7%	11.3%	12.9%	0.594	1.5 (0.5–3.9)
	3	3/51	0/80	3/131	5.9%	0%	2.3%	0.057	– ^2^
	5	1/51	1/80	1/131	0%	1.3%	0.8%	1	– ^2^
Type of location									
Exclusive	Genital	34/51	64/80	98/131	66.7%	80%	74.8%	0.101	0.5 (0.2–1.1)
Association	Genital-cutaneous	3/51	4/80	7/131	5.9%	5%	5.34%	1	1.2 (0.3–4.6)
Association	Oronasal	4/51	0/80	4/131	7.8%	0%	3.1%	0.021 *	– ^2^
Exclusive	Cutaneous	3/51	1/80	4/131	5.9%	1.3%	3.1%	0.299	4.9 (0.7–64.8)
Exclusive	Nasal	2/51	1/80	3/131	3.9%	1.3%	2.3%	0.559	3.2 (0.4–47.2)
Exclusive	Perineal	1/51	2/80	3/131	2%	2.5%	2.3%	1	0.8 (0.1–6.9)
Exclusive	Oral	0/51	1/80	1/131	0%	1.3%	0.8%	1	– ^2^
Exclusive	Bladder	0/51	1/80	1/131	0%	1.3%	0.8%	1	– ^2^
Association	Other	10/51	6/80	10/131	7.8%	7.5%	7.6%	0.322	1.7 (0.6–4.4)

* Significative association in Fisher’s exact test (*p* < 0.05). ^1^ Odds ratio considered Male as referent category (exposed). ^2^ In these binary outcomes in which the Male or Female groups had no occurrences of the outcome, calculating the odds ratio was not appropriate.

**Table 3:** Anatomical locations of canine transmissible venereal tumor in the Veterinary Hospital of the Universidade Federal de Minas Gerais, Belo Horizonte, Brazil (2012–2022).

Anatomical Locations	Cases in Male Dogs	Cases in Female Dogs	Total of Cases	*p*-Value	Odds Ratio ^1^
Genital	41/51	73/80	114/131	0.108	0.4 (0.2–1.0)
Cutaneous	9/51	6/80	15/131	0.094	2.6 (0.9–8.1)
Nasal	6/51	2/80	8/131	0.055	5.2 (1.2–25.9)
Oral	4/51	2/80	6/131	0.207	3.3 (0.7–17.8)
Lymph node	3/51	1/80	4/131	0.299	4.9 (0.7–64.8)
Perianal	2/51	2/80	4/131	0.642	1.6 (0.2–10.4)
Ocular	1/51	2/80	3/131	1	0.8 (0.1–6.9)

^1^ Odds ratio considered Male as referent category (exposed).

## Data Availability

Data from all cases are organized and presented in Supplementary Materials S1. Any other data may be provided upon request.

## Supplementary Materials

The following supporting information can be downloaded at https://www.mdpi.com/article/10.3390/ani15121675/s1, Supplementary Materials S1: The complete dataset of cases.

## Abbreviations

The following abbreviations are used in this manuscript:

CR	Complete response
CTVT	Canine transmissible venereal tumor
OR	Objective response
PR	Partial response
SD	Stable disease
